# An Integrated Approach for Identifying Wrongly Labelled Samples When Performing Classification in Microarray Data

**DOI:** 10.1371/journal.pone.0046700

**Published:** 2012-10-17

**Authors:** Yuk Yee Leung, Chun Qi Chang, Yeung Sam Hung

**Affiliations:** 1 Department of Electrical and Electronic Engineering, The University of Hong Kong, Hong Kong Special Administrative Region, China; 2 Department of Pathology and Laboratory Medicine, University of Pennsylvania, Philadelphia, Pennsylvania, United States of America; 3 Penn Center for Bioinformatics, University of Pennsylvania, Philadelphia, Pennsylvania, United States of America; I2MC INSERM UMR U1048, France

## Abstract

**Background:**

Using hybrid approach for gene selection and classification is common as results obtained are generally better than performing the two tasks independently. Yet, for some microarray datasets, both classification accuracy and stability of gene sets obtained still have rooms for improvement. This may be due to the presence of samples with wrong class labels (i.e. outliers). Outlier detection algorithms proposed so far are either not suitable for microarray data, or only solve the outlier detection problem on their own.

**Results:**

We tackle the outlier detection problem based on a previously proposed Multiple-Filter-Multiple-Wrapper (MFMW) model, which was demonstrated to yield promising results when compared to other hybrid approaches (Leung and Hung, 2010). To incorporate outlier detection and overcome limitations of the existing MFMW model, three new features are introduced in our proposed MFMW-outlier approach: 1) an unbiased external Leave-One-Out Cross-Validation framework is developed to replace internal cross-validation in the previous MFMW model; 2) wrongly labeled samples are identified within the MFMW-outlier model; and 3) a stable set of genes is selected using an L1-norm SVM that removes any redundant genes present. Six binary-class microarray datasets were tested. Comparing with outlier detection studies on the same datasets, MFMW-outlier could detect all the outliers found in the original paper (for which the data was provided for analysis), and the genes selected after outlier removal were proven to have biological relevance. We also compared MFMW-outlier with PRAPIV (Zhang *et al.*, 2006) based on same synthetic datasets. MFMW-outlier gave better average precision and recall values on three different settings. Lastly, artificially flipped microarray datasets were created by removing our detected outliers and flipping some of the remaining samples' labels. Almost all the ‘wrong’ (artificially flipped) samples were detected, suggesting that MFMW-outlier was sufficiently powerful to detect outliers in high-dimensional microarray datasets.

## Introduction

Classification is one of the major goals in microarray data analysis [1]–[7]. However, the quality of classifier depends critically on the correct labelling of the training data [8]. There are chances that some samples in a microarray data are given wrong class labels (due to subjective labelling, imperfectness in experiments or heterogeneity of data [9]. The presence of such mislabelled samples, even a small number of them, could severely degrade the performance of the classifier [8]. As reported in different studies using an unbiased validation model, perfect leave-one-out cross-validation (LOOCV) accuracies cannot be achieved in many microarray datasets [10]–[11] even though many gene selection tools have been combined with classifiers of different natures in various experiments. This suggests something wrong about these datasets which may be caused by the presence of wrongly labelled samples. We call these samples ‘outliers’. Their existence can only degrade the classification performance of any model. Previous work has reported the adverse impact of mislabelled samples on the performance of classification [8]. If no outlier detection and removal process is done either prior to or in conjunction with gene selection and classification, results obtained from the classification task can be seriously affected by the presence of these outliers. Consequently a promising outlier detection algorithm is essential for the microarray data analysis process.

Gene selection is another major goal in microarray data analysis; it is not only necessary for efficient classification, but also important for biomarker identification [12]–[14]. For microarray classification problems, evaluation on stability of gene sets is often neglected. Concern has recently been expressed regarding the fact that different studies reveal different gene sets for predicting the prognosis of breast cancer [15]–[16]. It is crucial to check whether the selected gene sets are stable or not, as a concise and stable gene set is easier to interpret. Besides, as the selected genes will be used for prognosis, a small set of genes is much cheaper and easier to be applied to large-scale dataset than long gene lists. The stability of selected genes refers to whether the same set of genes is chosen when perturbation of data occurs. Ideally, if only a small portion of training samples in two datasets is different, the sets of genes selected from these two datasets should not vary significantly. If large variations in selected gene sets are observed, this signifies something unusual among the samples in the data [17]. Note that stability only indicates the sensitivity of the gene selection algorithm with respect to perturbation of data, and does not necessarily have a bearing on the performance of selected genes. Various studies have proposed different ways of ‘stabilizing’ the gene selection process [18]–[19]. As different sets of genes are selected corresponding to different perturbations, the selected genes can be ranked by their frequency of selection. A gene is most certain to be relevant to the classification task if it is selected most of the time. Gene set stability is often evaluated by LOOCV to see how consistent the selected gene set is when different samples are left out. It is possible that the selected gene sets are fairly stable, except when the left-out sample is an outlier. This is because for all training datasets containing the outlier, the outlier affects each of these training datasets in a similar way resulting in a ‘stable and consistent’ set of genes to be selected. But once the outlier is removed from the training dataset, its influence is lost and so the selected gene set may be quite different. Hence, it is crucial that outliers be removed for gene stability to be taken as a useful measure.

Outlier detection is a process to search for samples that do not obey the general rules of the majority portion of the data of the same class. Many outlier detection algorithms have been proposed, yet most of them [20]–[22] attempt to detect outliers by computing the distances in the full dimensional space. As microarray data is of high dimensional space, and due to the sparse nature of distance distributions, the concept of similarity may not be meaningful [20]–[21]. Since outlier detection algorithms developed for other domains are not suitable for microarray data, tailor-made outlier detection methods for detecting wrong-labelled samples are proposed. Furey *et al.* applied SVM on microarray datasets with reduced set of genes. Samples which have been consistently misclassified in all tests are identified as suspects [9]. Kadota *et al.* proposed a method based on Akaike's Information Criterion to detect outliers in the colon data [23]. In the study conducted by Lu *et al.*, outliers are detected using support vector machine (SVM) in a re-validation framework [24]. Zhang *et al.* introduced the misclassification probability which is estimated for each sample in the training set [25]. Unger and Chor developed a method for finding all pairs of genes that induce a linear separation of the two sample classes. If no gene pairs can separate the two classes distinctly, then the dataset contains outliers [26]. In the study by Malossini *et al.* [8], two algorithms are designed for detecting possible mislabelled samples: a Classification-stability (CL-stability) algorithm and a Leave-One-Out-Error-sensitivity (LOOE-sensitivity) algorithm. The CL-stability algorithm evaluates the stability of classification of a sample by perturbing a small amount of samples, whereas LOOE-sensitivity is based on the idea that the classification accuracy should be improved after flipping the label of a mislabelled sample. In 2011, Zhou *et al.* modified the CL-stability approach. Their goal was to detect outlier samples and automatically correct them, and their proposed method was called Fast Outlier Samples Detection (FOSD) [27].

The aims in all the above studies are to design an outlier detection algorithm on their own. A distinctive feature of our proposed MFMW-outlier framework is that outlier detection is integrated within a proposed hybrid approach. This ‘three-in-one’ approach performs gene selection, classification and outlier detection simultaneously, which is particularly suitable for high-dimensional microarray datasets.

## Materials and Methods

### Datasets

Our aim was to identify any wrongly labelled samples present in a high dimensionality data, such as microarray. Six benchmark binary-class datasets on cancers were selected for evaluation using the algorithm proposed. With the help of synthetic datasets, the effectiveness of MFMW-outlier could be demonstrated despite the absence of ground truth information for which samples are outliers.

1. **Microarray datasets.** The six chosen binary-class datasets, all generated using Affymetrix chips, were: *LEU*, *COL*, *BRE*, *LYM*, *PROS* and *LUNG*. They were pre-processed according to the instructions published in the original paper. In addition, each sample was normalized to have mean zero and unit variance. Table 1 summarizes the data we used.
2. **Synthetic datasets.** Synthetic datasets were more reliable as the class labels for all samples were known. Experimental results obtained from these datasets could therefore reflect the true performance of the proposed algorithm. In a recent study on detecting outliers in microarray data [25], artificial datasets were generated as part of their experiments. The number and characteristics of samples and features included in our synthetic datasets were the same as theirs. Although microarray datasets may have different characteristics, e.g. varied number of genes, varied proportion of samples in each class or varied number of classes, our main objective here is to compare MFMW-outlier with the performance in [25] using the same datasets. Each of our synthetic binary-class datasets contained 30 samples, with equal number of samples in each class. Each of the samples was given a class label of +1 or −1. A total of 10,000 features (typical number of genes on microarray) were randomly generated, of which 5 were discriminating ones created based on Gaussian Distributions. μ and σ are the mean and standard deviations of the discriminatory features. For class 1, μ = −3 and σ = 1 while for class 2, μ = −3 and σ = 3. The remaining features were generated as Gaussian noise. A total of 3 different synthetic datasets were created based on the above characteristics. They differed only in the number of mislabeled samples present: 4 (Test 1), 6 (Test 2) and 10 (Test 3). These corresponded to Test 1–3 generated by Zhang *et al.* [25]. Table 2 summarizes the synthetic datasets we used.

**Table 1:** Summary of the six binary-class microarray datasets.

Dataset	No. of samples	No. of genes	References
***LEU***	38 ALL, 34 AML	7129	[1]
***COL***	40 cancer, 22 normal	2000	[2]
***BRE***	25 ER+, 24 ER−	7129	[3]
***LYM***	58 DLBCL, 19 FL	7129	[4]
***PROS***	50 normal, 52 cancer	12000	[5]
***LUNG***	150 ADCA, 31 MPM	12600	[6]

**Table 2:** Design of our synthetic datasets.

Dataset	No. of samples	No. of genes	No. of mislabeled samples
**Test 1**	15 Class 1, 15 Class 2	10000	4
**Test 2**	15 Class 1, 15 Class 2	10000	6
**Test 3**	15 Class 1, 15 Class 2	10000	10

### Methods

Our proposed integrated MFMW-outlier approach is built on top of the MFMW model proposed in [28]. However, to address the limitations, we propose here three major modifications to MFMW, and will refer to the new model as N-MFMW:

1. The optimal number of genes selected in the multiple-wrapper step in MFMW-outlier is determined using an L1-norm SVM, whereas those selected in earlier approach was based on a threshold, i.e. a fixed number of genes.
2. The entire gene selection and classifier procedure of MFMW-outlier is built within a fully unbiased LOOCV framework (i.e. external LOOCV) in our present approach, as opposed to applying LOOCV only to the classification part only in [28].
3. Identifying wrongly labelled samples is made possible through the use of external LOOCV, as each sample is left out completely from start, including the gene selection process.

Since the present approach is based on the MFMW model developed in [28], in the following we only discuss the new features introduced here beyond the framework already developed in [28]. We refer to this new model as N-MFMW. Interested readers are referred to the paper [28] for more details on the original MFMW.

#### 1. MFMW Model under External LOOCV

**Figure 1:**
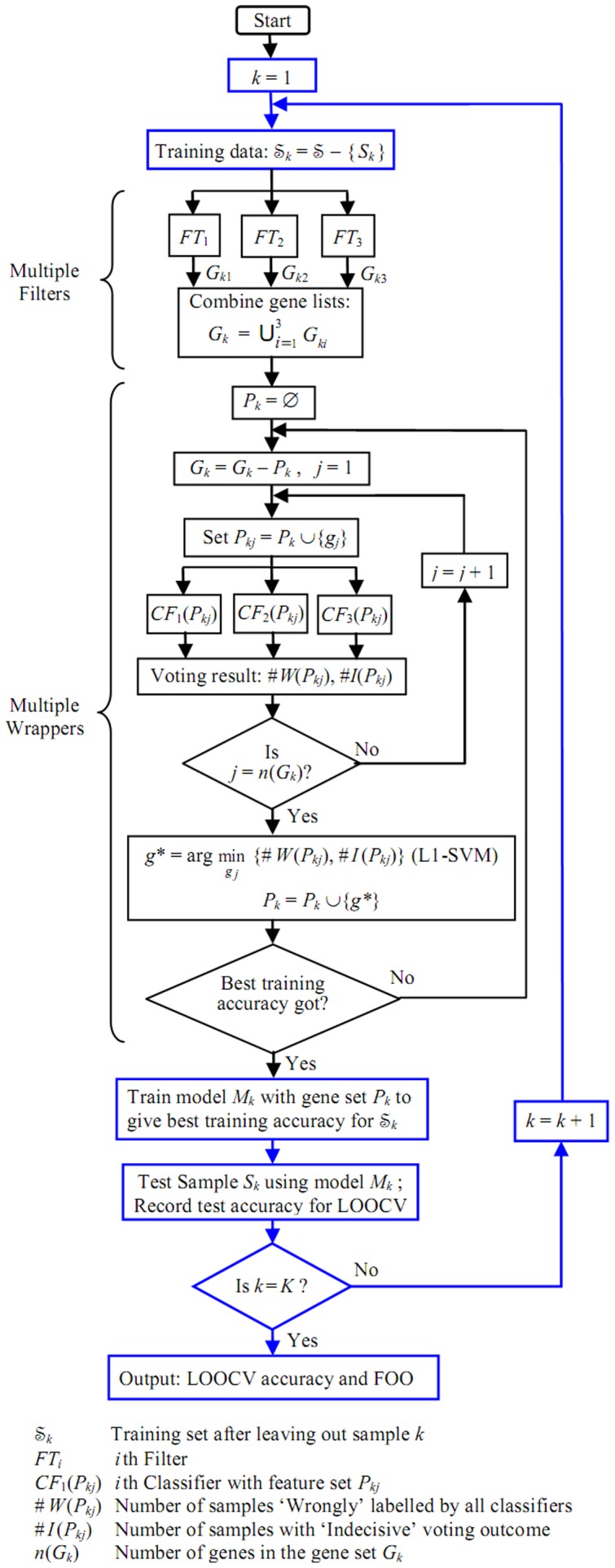
N-MFMW model in an external LOOCV framework.

(This is a modification of the original MFMW). Let 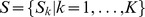 be a dataset of samples. The N-MFMW model in an external LOOCV framework is illustrated in Figure 1. LOOCV is performed in the outermost loop (together with the boxes highlighted in blue), whereby each sample 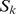 is left out in turn before N-MFMW is applied to the training set 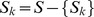. Note that all subsequent symbols with subscript 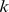 refer to data generated after leaving out sample 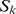.

#### Multiple-Filter part

The same three filters were used as in MFMW. One hundred and fifty genes are selected by each filter and the three gene lists are then combined by taking their union.

#### Multiple-Wrapper part

The same three wrappers were used in MFMW. Please refer to [28] for details.

#### L1-SVM for incremental gene selection

(This is a modification of the original MFMW) Suppose the set of samples 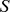 are represented by points 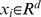 where each 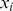 belongs to one of two classes with label 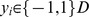. An SVM is constructed by computing a classifier function 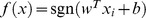, where the parameters 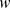 and 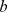 are determined by optimization:
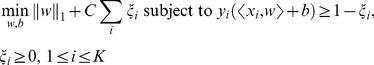
(1)The parameter 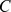 is a cost parameter and is provided as an input. In the above optimization, we seek to minimize 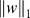 instead of the usual L2-norm 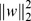 in traditional SVM. Minimizing 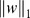 tends to give sparser solutions, which imply better dimension reduction yielding classifiers of greater robustness [29].

In N-MFMW model, the number of genes selected at each level of the wrapper part is ‘optimized’ using L1-SVM. Instead of taking one/all gene(s) at the same level, the number of genes selected is based on the 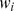 scores outputted by the L1-SVM. The reason why L1-SVM is chosen is due to its singularity nature. This nice property allows the automatic selection of relevant genes with respect to class labels of samples when there are several highly correlated genes. The larger the 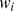 score, the more informative and less redundant the gene is, as compared to other genes of the same level. A cut-off threshold is required for choosing a certain number of genes. This is determined by finding the largest difference between these 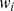 values. By picking only a few genes and removing the rest, L1-SVM selects a small subset of genes from all the genes that have the same number of ‘
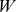
’ and ‘
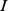
’ [30]. Instead of selecting a pre-defined number of genes (as proposed in [28], [31]), the final number of selected genes is determined by 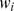 values in N-MFMW, which is data dependent.

#### External LOOCV and the final classification model

The entire N-MFMW process is repeated for every sample 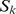. At the 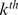 round of the LOOCV, after the incremental gene selection process stops, we obtain the final gene set 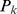 and the corresponding classification model 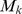, which is composed of the feature set 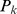, the group of classifiers used as multiple wrappers, and the voting scheme. The final model is trained again using final gene set 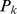 and the sample set 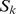 to give the best training accuracy. The trained model 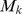 is then used to test the left-out sample 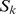, yielding the test accuracy for 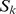. Finally, the LOOCV accuracy is computed as the percentage of correctly classified test samples, and the gene stability measure Frequency Of Occurrence (FOO) of a particular gene is calculated as the number of times that gene is found on the list 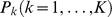 outputted by the N-MFMW algorithm, divided by 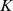.

#### 2. Outlier Detection part

**Figure 2:**
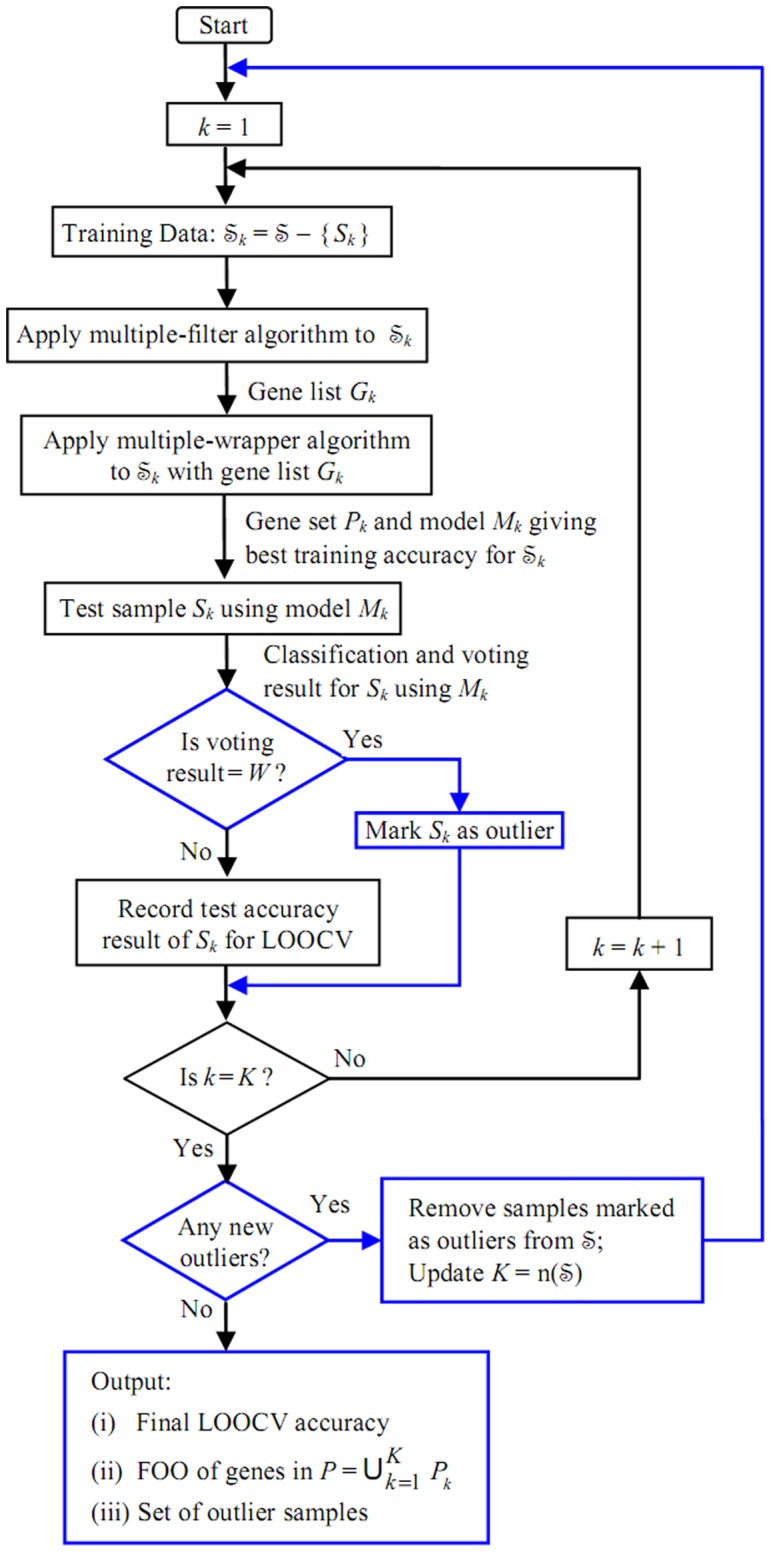
MFMW-outlier: Integrating outlier detection into N-MFMW model with external LOOCV.

We now propose to incorporate outlier detection into the N-MFMW model with external LOOCV, as shown in Figure 2, which may be regarded as an expansion of Figure 1 by introducing additional steps (highlighted in blue) for outlier detection. As shown in Figure 2, a test sample 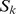 is marked as an outlier if it is misclassified by all three classifiers of the model 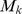 trained by N-MFMW based on 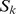. This is a highly convincing condition as it requires all classifiers, each based on all samples other than the one under testing, to agree unanimously that 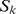 ‘acts’ as if it has a label of the opposite class. Such samples marked as outliers are removed only after one complete cycle of LOOCV is performed. Since the removal of any outlier(s) may have a significant impact on gene selection and hence the N-MFMW training process, the entire LOOCV exercise has to be repeated after outliers are removed.

Finally, the LOOCV accuracy and the gene stability measure FOO can be computed based on a reduced dataset that has been cleansed of outliers. The set of genes can then be ranked according to their FOO scores from the largest to smallest, with a cut-off threshold set for FOO values. The complete algorithm that integrates all three components of gene selection, optimization of classification accuracy and outlier detection, is given below.

MFMW-outlier – Outlier Detection under N-MFMW with External LOOCV:

1. Start with 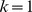.
2. Define Training set 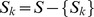.
3. Apply ‘Multiple Filters’ algorithm to 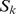 to get filtered gene list 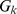; apply ‘Multiple Wrappers’ algorithm to get gene set 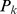 and train model 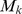 to give best accuracy for 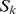. Record 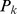 for computation of gene stability measure FOO.
4. Apply *M_k_* to test sample 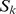; if the classification result is ‘
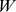
’, mark 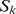 as an outlier, otherwise the classification result is recorded for computation of LOOCV accuracy.
5. Repeat steps 2–4 for all 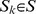.
6. If there are no samples marked as outliers, proceed to the next step, otherwise remove all samples 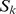 marked as outliers from 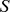, update 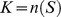, and repeat steps 1–5.
7. Output:
  - LOOCV accuracy based on test results for each 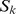;
  - FOO of genes in 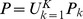;
  - Set of outlier samples.

The proposed MFMW-outlier is a significant modification of the MFMW model proposed in [28], [31] in that it represents a ‘three-in-one’ approach integrating all three components of gene selection, classification and outlier detection in an unbiased external LOOCV framework. Like MFMW, the underlying idea of N-MFMW models is to first use multiple filters with complementary characteristics to select genes, which are then merged to provide a filtered subset of (several hundreds of) genes. Different filters select genes with different statistical properties across the classes under study. Therefore the use of multiple filters of different natures ensures that potential biomarkers are unlikely to be screened out by one specific filter criterion in an initial stage. After gene screening by multiple filters, multiple wrappers are used for incremental gene selection based on training accuracy. The use of multiple wrappers, together with a unanimous voting scheme, is for enhancing the robustness of the training accuracy by means of consensus. Though one of the toughest issues for the wrapper methods is computational complexity [32], our approach handles this problem in two ways: 1) restricting the number of genes to be included, largely by the use of multiple filters, which is in turn determined by the number of genes picked up by each filter (in our experiment we used 20); 2) choosing a simple classifier: e.g. *k*-NN will perform much faster than neural network, genetic algorithms or other classifiers that require optimization of lots of parameters.

An external LOOCV is then performed to evaluate the performance of the classifier. More importantly, the use of external LOOCV in the N-MFMW framework allows a natural outlier detection and gene set stability evaluation. LOOCV is chosen instead of other model estimation methods (like bootstrapping) as a score can be assigned to each sample specifically for the determination of whether it is an outlier or not. This score can be easily obtained if only one sample is left out each time. When multiple samples are left out (as in the case of bootstrapping or other cross-validation tools), each time certain portions of samples are used in testing, and the final score for outlier detection will be dependent on the results from several runs. There are two disadvantages of doing so: 1) including several samples together as a test set will obscure the signal of an outlier (if any); 2) additional steps are required to combine the scores from different runs.

Though cross validation should be normally performed to evaluate the performance of a classifier, in previous microarray studies, the most common misuse of validation involves selecting genes using the full dataset, called internal cross-validation, rather than performing gene selection from scratch within each loop of the cross-validation [33]. MFMW was built upon this and this is the major limitation [28], [31]. Internal cross-validation may favourably bias the performance of the classifier, as the training and testing parts of the dataset are not independent of each other [34]. Studies have shown that this may lead to very optimistic estimates of the error rate [35]–[36]. To address this, we employ external LOOCV in N-MFMW where gene selection is performed without the benefit of knowledge of the test sample.

## Results and Discussion

### Results on six microarray datasets

We first applied MFMW-outlier on the six microarray datasets. These selected datasets are the most ‘well-studied’ ones in the microarray community. The first three datasets used in our current study [8], [25]–[27], [37] have also been used in other newly proposed outlier detection methodologies, and for comparison purpose, they are included here. For most microarray datasets in public domains, there is no available information as to which of the samples might possibly be an outlier. The lack of ground truth makes these less suitable for the present study.

Table 3 summarizes all the removed outliers in each iteration in the external LOOCV. For five out of the six microarray datasets we worked on, different number of outliers were removed in each iteration. For *LUNG* data, no outlier was detected.

**Table 3:** Sample(s) removed as outliers in each iteration of MFMW-outlier for all the six microarray datasets.

Dataset	Iteration	Samples left (#)	Suspected outlier (sample ID)
***LEU***	1^st^	72	66
	2^nd^	71	*NIL*
***COL***	1^st^	62	T33, T36, T37, N20
	2^nd^	58	T2, T30
	3^rd^	56	N2, N8, N18
	4^th^	53	*NIL*
***BRE***	1^st^	49	Marks206, Marks213, Nevins24, Nevins26, Marks219, Marks220
	2^nd^	43	Marks204, Marks216, Nevins21
	3^rd^	41	*NIL*
***LYM***	1^st^	77	DLBC26, FSCC12, FSCC13, FSCC16
	2^nd^	73	DLBC29, DLBC36 and FSCC18
	3^rd^	70	*NIL*
***PROS***	1^st^	102	N35_normal, N38_normal, T39_tumor, T49_tumor, T54_tumor
	2^nd^	97	N06_normal, T17_tumor, T37_tumor
	3^rd^	94	*NIL*
***LUNG***	1^st^	181	*NIL*

Comparison with other proposed outlier detection methodologies on microarray datasets were made [8], [25]–[27], [37]. For *LEU* data, the only outlier being detected in every algorithm is Sample 66. Tables 4 and 5 compare the outlier detection results using different methods on the other two datasets: *COL* and *BRE*.

**Table 4:** List of outliers detected by different proposed methods on *COL*.

	Original	CL-Stability	PRAPIV	FOSD	MFMW-outlier
Sample ID.	[2]	[8]	[25]	[27]	NA
T2	Y	Y	-	Y	Y
T30	Y	Y	Y	Y	Y
T33	Y	Y	Y	Y	Y
T36	Y	Y	Y	Y	Y
T37	Y	-	Y	Y	Y
N8	Y	-	Y	Y	Y
N12	Y	-	-	-	Y
N34	Y	Y	Y	Y	Y
N36	Y	Y	Y	Y	Y
Others	NA	N2, N28	N2, N28	N2, N28	NA

**Table 5:** List of outliers detected by different proposed methods on *BRE*.

	Original	CL-Stability	PRAPIV	FOSD	MFMW-outlier
Sample ID.	[3]	[8]	[25]	[27]	NA
Nevins21	Y	-	-	-	Y
Nevins24	Y	Y	-	Y	Y
Nevins26	Y	Y	Y	Y	Y
Marks204	Y	Y	Y	Y	Y
Marks206	Y	-	-	-	Y
Marks213	Y	-	Y	Y	Y
Marks216	Y	Y	Y	Y	Y
Marks219	Y	Y	-	Y	Y
Marks220	Y	-	-	-	Y
Others	NA	47	19	NA	NA

In both Tables 4 and 5, MFMW-outlier was the only method that can detect all the outliers (mislabelled samples) claimed in the original paper (for which the datasets were provided for analyses). This shows MFMW-outlier was the most consistent with the ‘ground truth’. For other methods, both false positives and false negatives were found in both *COL* and *BRE* datasets.

At the end of MFMW-outlier, a set of stable genes was selected. Table 6 shows the gene sets chosen for each of the six datasets. Genes selected by MFMW-outlier were quite different from other published results including our MFMW model [28]. This is due to the fact genes in Table 6 were selected after all susceptible outliers were removed, which means they are of better confidence as they are not affected by the possible contaminations underlying the samples. Also the genes selected here are from a fully unbiased cross-validation model. Selected genes from Table 6 with biological significances in the published literatures are discussed as follows:

- LEU
  - CST3 is related to AML [38].
  - MGST3 was linked indirectly with GSTM1 according to BioGraph [39]. Polymorphism in GSTM1 was shown to have effect on the ALL patients. [40].
  - By BioGraph [39], PSMB8 was indirect related to a number of genes (PTPN1, BAD, PRAME, BIRC2, CFLAR and MLL) causing AML and it was a gene target for a study using high-throughput gene mutation analysis in AML [41].
  - The relationship of MYB with acute leukemias has been shown [42].
- COL
  - VIP has been shown to regulate the growth of colonic adenocaricinoma cells [43].
- BRE
  - DSC3 expression was down-regulated in more than half of breast cancers [44].
  - ETV1, also known as ER81, was shown to be collaborated with the oncoprotein HER2/Neu to activate Smad7 transcription in breast cancer cell lines [45].
- LYM
  - Transgenic mice overexpressing HMGA1 gene was shown to develop natural killer cell lymphomas [46] and by BioGraph [39], HMGA1 was directly related to mir16-1, which is a lymphoma causing miRNA [47].
  - The updated annotation for JTV-1 is AIMP2 gene, which is shown to have protein interaction with RARS gene, which has direct relationship to mir16-1 by BioGraph [48].
- PROS
  - HPN is a potentially important candidate gene involved in prostate cancer susceptibility [49].
  - NELL2 mRNA expression was predominantly localized in basal cells of the epithelium in situ hybridization analysis of hyperplastic prostate specimens [50]
- LUNG
  - According to BioGraph [39], KLK3 is interacting with PTHLH, which is a disease causing gene for non-small cell lung carcinoma [51].
  - By Biograph [39], PTRF is transcriptionally regulating ERCC6 gene, which is a disease causing gene of lung carcinoma [52].
  - Similarly, SERPINH1 is interacting with CD9 gene, which is a disease causing gene of non-small cell lung carcinoma [53].

**Table 6:** Final stable set of genes (gene symbols shown) obtained from performing MFMW-outlier (after removal of outliers) on six microarray datasets.

*LEU*	*COL*	*BRE*	*LYM*	*PROS*	*LUNG*
CST3	VIP	UBE3A	HLA-A	HPN	KLK3
MGST3	GSTM4	DSC3	HMGA1	LMO3	PTRF
PSMB8		ETV1	JTV-1	NELL2	SERPINH1
MYB			ENO1	MTHFD2	
			TCRB		

### Results on three synthetic datasets

Besides microarray datasets, MFMW-outlier was also evaluated upon using three synthetic datasets (Tests 1–3 in Table 2). We compared our results with PRAPIV [25]. To determine the ability of detecting the outliers of the two algorithms, mean precision and recall values were used for evaluation and were summarized in Table 7. Precision is defined as the portion of true outliers in all the detected outliers, while recall is defined as the portion of detected outliers in all the ground truth outliers.

**Table 7:** Comparison of the mean precision and recall values on the synthetic datasets.

	Test1		Test 2		Test 3	
	PRAPIV	MFMW-outlier	PRAPIV	MFMW-outlier	PRAPIV	MFMW-outlier
**Precision**	83.41%	98.15%	76.44%	96.39%	54.84%	96.86%
**Recall**	91.50%	98.91%	83.33%	96.77%	59.00%	97.34%

Table 7 shows that MFMW-outlier gave much better precision and recall values across all three synthetic datasets, demonstrating the robustness of MFMW-outlier. Regardless of the number of outliers present in the data, MFMW-outlier could detect almost all of them.

### Results on artificially flipped microarray datasets

It's almost impossible to know which samples are wrongly labelled in typical microarray datasets. According to microarray studies on Colon cancer [2] and Breast cancer [3], the samples under column ‘Original’ in Tables 4 and 5 were identified as outliers with biological evidences. Similar to what Zhang *et al.* have performed [25], these two datasets were selected for constructing artificially flipped datasets. After removing the outliers under the ‘Original’ column, six samples were randomly selected from the reduced dataset and their class labels were flipped. We then applied MFMW-outlier to these datasets, with an aim to identify these six samples. Experiments were performed on each dataset (reduced-*COL* and reduced-*BRE* respectively) 50 times. We report how accurate we were able to detect the six artificially labelled samples, with comparison to PRAPIV [25], in terms of mean precision and recall values as summarized in Table 8. The results demonstrated that MFMW-outlier yielded much better precision and recall values for both flipped datasets, as compared to PRAPIV.

**Table 8:** Comparison of the mean precision and recall values on flipped microarray datasets.

	Reduced-*COL*		Reduced-*BRE*	
	PRAPIV	MFMW-outlier	PRAPIV	MFMW-outlier
**Precision**	71.61%	96.87%	88.44%	95.49%
**Recall**	87.65%	98.28%	91.46%	94.54%

If an algorithm reports *N* outliers, denote *p* as precision and *r* as recall, we expect that there are 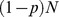 false positives (FP) and 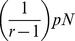 false negatives (FN). Assume that these mean precision and recall obtained from flipped microarray datasets also apply to the real microarray dataset, through simple calculation, for MFMW-outlier there should be 0.28 FP and 0.15 FN in *COL* and 0.41 FP and 0.50 FN in *BRE*, which corroborate with the results shown in Tables 4 and 5, demonstrating that the excellent result obtained for the two real datasets are highly reliable rather than just by chance. As a comparison, for PRAPIV there should be 2.56 FP and 0.91 FN in *COL* and 0.58 FP and 0.41 FN in *BRE*, which is over optimistic when compared to the real values shown in Tables 4 and 5, further demonstrating the superior robustness of MFMW-outlier.

### Effects of filters and wrappers on MFMW-outlier model

We are also interested in how classification performance changes when different number of filters and wrappers are used in our MFMW-outlier model. Consider varying the number of filters in the model. If too few filters are used, inadequate genes of dissimilar characteristics are selected. If too many filters are used, some of the selected genes across the different gene lists are redundant in nature. To investigate how many filters should be included, we check if the biological significant genes discussed in the previous session are all present in all gene lists produced by different filters. The gene lists are obtained by setting *n* = 200 genes for each filter. Table 9 below shows the presence (Y) or absence (N) of each gene within the top ranked 200 genes selected by the three filters we used.

**Table 9:** Presence or absence of biological significant genes as selected by different filters (n = 200).

		SNR	TS	AUC
***LEU***	**CST3**	Y	N	Y
	**MGST3**	Y	Y	Y
	**PSMB8**	Y	Y	N
	**MYB**	Y	Y	Y
***COL***	**VIP**	Y	Y	Y
***BRE***	**DSC3**	Y	Y	Y
	**ETV1**	N	N	Y
***LYM***	**JTV-1**	Y	Y	N
	**HMGA1**	N	N	Y
***PROS***	**HPN**	Y	Y	Y
	**NELL2**	N	Y	Y
***LUNG***	**KLK3**	Y	Y	Y
	**PTRF**	Y	Y	Y
	**SERPINH1**	Y	Y	Y

The following conclusions can be drawn from results in Table 9:

- SNR and TS select almost the same set of genes, except for CST3 in LEU, and hence if SNR is not included as one of the filter, this gene would be missed out
- Similar for the case of NELL2 in PROS, and so TS is an important filter.
- There are two genes: ETV1 in BRE and HMGA1 in LYM which are only selected by AUC, but not by the other two filters. This suggests that AUC is an important filter.

Next, we investigate if the number of wrappers (and if possible, choice of wrappers) used in the MFMW-outlier model would result in variations in the classification performance of the model. By the basic idea of the multiple wrapper approach, there has to be more than two wrappers. On the other hand, using too many wrappers of similar nature does not provide more information for the decision process. Therefore, the number of wrappers employed in the following experiment varies from two to four. In addition to the three wrappers we used, the extra wrapper chosen is Naïve Bayes (NB). This wrapper is selected because it is one of the most popular classifiers used in microarray experiments. In each experiment, 200 genes are first selected by each of the three filters. Experiments with different number of wrappers are then performed on the LYM dataset.

Using genes selected by three filters, Table 10 shows the experimental results obtained from MFMW-outlier models consisting of two wrappers. As four wrappers are available, there are a total of _4_C_2_ = 6 experiments. For each experiment, we select the final gene sets with the smallest values of ‘
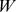
’ and ‘
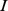
’. Very often there are multiple gene sets of this characteristic. These set(s) of genes are then evaluated and biased LOOCV accuracies are obtained. Only when the gene set gives a perfect LOOCV accuracy is its result recorded in the table. ‘# of genes’ is the size of gene set selected by each MFMW-outlier models. ‘# of subsets giving perfect LOOCV accuracy’ is the number of gene sets that output a perfect biased LOOCV accuracy. From Table 10, the best sets of wrappers (of size two) are ‘WV+*k*-NN’, ‘WV+SVM’ and ‘WV+NB’. All three models use an equally small set of eight genes for perfect LOOCV performance. Although the two models ‘*k*-NN+SVM’ and ‘*k*-NN+NB’ both select eight genes in the final gene set, the variations in terms of selected genes are too large to allow one to decide on which gene set should be chosen finally. Therefore they are not selected as the best models.

**Table 10:** MFMW-outlier results obtained from using three filters (n = 200) and two wrappers for LYM dataset.

Wrappers	# of genes	# of subsets
WV+*k*-NN	8	1
WV+SVM	8	1
WV+NB	8	2
*k*-NN+SVM	8	20
*k*-NN+NB	8	18
SVM+NB	6	0

Using genes selected by three filters, Table 11 shows the experimental results obtained from MFMW-outlier models built by three or four wrappers. There are altogether _4_C_3_ = 4 experiments built using three wrappers. The best set of wrappers of size three is ‘WV+*k*-NN+SVM’, which is the same as the one we presented earlier. Also, MFMW-outlier models built by using four wrappers are not as good as that built by using three wrappers. Hence, for the LYM dataset, using three wrappers for the MFMW-outlier is most appropriate. Similar results (details not shown here) have been obtained for other datasets. Note that the size of gene set selected using MFMW-outlier based on three wrappers is six, which is smaller than that (i.e., eight) selected using two wrappers. As a smaller set of genes is able to give the same biased LOOCV accuracy, we would recommend using the three wrappers ‘WV+*k*-NN+SVM’ in MFMW-outlier model.

**Table 11:** MFMW-outlier results obtained from using three filters (n = 200) and three/four wrappers for LYM dataset.

Wrappers	# of genes	# of subsets
WV+*k*-NN+SVM	6	1
WV+*k*-NN+NB	6	2
WV+SVM+NB	6	4
*k*-NN+SVM+NV	6	2
WV+*k*-NN+SVM+NB	8	3

### Extension for multiclass datasets

The proposed method can be extended to the case of multiclass setting involving datasets with more than two classes. The straight forward way is to build a classification model for each class that separates this particular class from the remaining classes. This is a one-versus-all (OVA) classification approach. Another possibility is to train the classification model for every pair of classes in the multiclass dataset. This is a one-versus-one (OVO) classification approach. The challenge for the latter method is that an outlier detected in an OVO model may not be a true outlier, as it may be a sample that belongs to a class other than the two classes used for building the OVO model. We would therefore recommend building MFMW-outlier in an OVA manner. Other than this, integrating MFMW-outlier with other multiclass methods like error-correcting-codes approach will require more efforts, as such approaches require the design of codes for classification beyond the usage of just binary-class classifiers.

## Conclusions

The main contribution of this paper is to integrate outlier detection into an existing hybrid model, while making two significant modifications to the hybrid model to address issues on optimal gene selection and the problem of bias in internal cross validation. The new ‘three-in-one’ MFMW-outlier model can handle gene selection, sample classification and outlier detection simultaneously. MFMW-outlier was evaluated using both microarray and synthetic datasets. All results showed that we were able to detect the outlying samples present in high dimensional data. When comparing with ‘ground truth’ obtained from original paper, we were able to detect all the mislabelled samples, whereas other methods may result in some FP and FN. The fact that the selected genes were biologically confirmed was a strong indication that we have removed the wrong samples correctly.

To conclude, we have demonstrated the feasibility of integrating outlier detection into a hybrid model. The model is shown to have very high robustness with respect to the number of outliers in the dataset. We have implemented the proposed algorithm in MATLAB® and the software is available at http://people.pcbi.upenn.edu/~yyee/MFMW-outlier/.
